# Optimizing Prostate Biopsy Pathways: Integrating MRI–Targeted, Systematic Sampling, and Clinical Judgment in the PSA-Era

**DOI:** 10.3390/diagnostics16030389

**Published:** 2026-01-26

**Authors:** Catalin Andrei Bulai, Razvan Andrei Stoica, Adrian Militaru, Ana Maria Andreea Punga, Razvan Ionut Vaduva, Razvan Dragos Multescu, Cristian Mares, Cosmin Victor Ene, Bogdan Florin Geavlete

**Affiliations:** 1 Faculty of General Medicine, “Carol Davila” University of Medicine and Pharmacy, 020021 Bucharest, Romania; catalin.bulai@umfcd.ro (C.A.B.); adrian.militaru@drd.umfcd.ro (A.M.); ana-maria-andreea.punga@drd.umfcd.ro (A.M.A.P.); razvan.multescu@umfcd.ro (R.D.M.); cristian.mares@drd.umfcd.ro (C.M.); cosmin.ene@umfcd.ro (C.V.E.); bogdan.geavlete@umfcd.ro (B.F.G.); 2 Department of Urology, “Saint John” Emergency Clinical Hospital, 042122 Bucharest, Romania; stevad196214@gmail.com; 3 Department of Surgery, “Saint John” Emergency Clinical Hospital, 042122 Bucharest, Romania

**Keywords:** prostate cancer, multiparametric MRI, PSA density, MRI-targeted biopsy, systematic biopsy, combined biopsy, clinically significant prostate cancer, diagnostic pathways

## Abstract

Prostate cancer diagnostics have evolved substantially with the integration of multiparametric magnetic resonance imaging (mpMRI), refined prostate-specific antigen (PSA) metrics, and targeted biopsy techniques. While mpMRI has become a central gatekeeper in biopsy decision-making, it is not infallible. Clinically significant prostate cancer may therefore remain undetected, particularly in patients with elevated PSA density, adverse PSA kinetics, or MRI-occult disease. This narrative review synthesizes contemporary evidence on PSA interpretation, mpMRI performance, and biopsy strategy selection, highlighting the limitations of single-parameter approaches. We discuss the diagnostic yield and clinical implications of targeted, systematic, and combined biopsy techniques, emphasizing scenarios in which systematic sampling remains necessary despite negative or equivocal imaging findings. Emerging data support combined targeted and systematic biopsy as the most robust strategy for maximizing the detection of clinically significant disease while limiting overdiagnosis in most biopsy-naive and high-risk patients. By integrating PSA dynamics, prostate volume, imaging findings, and individual risk profiles, a structured, risk-adapted diagnostic pathway can be achieved. The proposed framework is intended as a conceptual, expert-derived clinical aid to support risk-adapted decision-making. It should be interpreted alongside established guidelines, and prospective validation in future studies is warranted.

## 1. Introduction

Prostate cancer remains one of the most prevalent malignancies worldwide, and its diagnostic pathway has undergone substantial transformation with advances in imaging, biomarkers, and biopsy techniques [1,2]. Among these, multiparametric magnetic resonance imaging (mpMRI) has become a central component of contemporary risk stratification, enabling improved detection of clinically significant prostate cancer (csPCa) while reducing unnecessary biopsies [3,4]. In this review, csPCa is defined as International Society of Urological Pathology (ISUP) Grade Group 2 or higher, unless otherwise specified [5].

Even when performed and interpreted under high-quality conditions, mpMRI has clear blind spots. A negative examination reduces the probability of csPCa, but it does not eliminate it, particularly in men with non-low baseline risk or adverse prostate-specific antigen (PSA)-derived parameters [6]. In routine practice, this is exactly where decision-making becomes difficult: imaging must be weighed against prostate-specific antigen density (PSAD), PSA kinetics, prostate volume, and the overall clinical picture, rather than being treated as a stand-alone gatekeeper.

Parallel to advances in imaging, serum prostate-specific antigen (PSA) remains central to early detection strategies, though its interpretation has become increasingly nuanced. Prostate-specific antigen levels are influenced by benign prostatic hyperplasia, inflammation, age, and transient physiological factors, limiting the reliability of isolated measurements [7]. Contemporary practice therefore emphasizes PSAD, PSA kinetics, and adjunctive biomarkers such as the Prostate Health Index (PHI) and 4Kscore, which enhance discrimination between csPCa and benign conditions, including prostatitis [8,9]. Fluctuating PSA patterns, disproportionate PSA elevation relative to prostate volume, and transient rises associated with pelvic symptoms often reflect inflammatory processes rather than malignancy [10]. Integrating PSA dynamics with mpMRI findings is essential to minimize unnecessary biopsies and patient anxiety.

Biopsy techniques have evolved accordingly. Magnetic Resonance Imaging (MRI)–ultrasound fusion biopsy has become increasingly accessible and improves localization and sampling of Prostate Imaging-Reporting and Data System (PI-RADS) 4–5 lesions, achieving higher csPCa detection rates than systematic biopsy alone [4]. Nevertheless, systematic transperineal sampling remains critical, as exclusive reliance on targeted cores may miss significant disease, particularly in multifocal or MRI-occult tumors [11]. As a result, international guidelines now recommend a combined targeted-plus-systematic approach for most biopsy-naive patients [12,13].

Despite a robust evidence base, several clinical scenarios remain challenging, including the management of PI-RADS 3 lesions, persistently elevated PSA with negative mpMRI, prostatitis mimicking malignancy, and ongoing diagnostic uncertainty after negative biopsy. These grey zones underscore the need for structured, integrated diagnostic algorithms [14,15].

A structured literature search was performed in PubMed and Scopus, focusing on predominantly English-language publications from the past decade. Search terms included combinations of “prostate-specific antigen”, “PSA density”, “PSA velocity”, “multiparametric MRI”, “PI-RADS”, “MRI-targeted biopsy”, “systematic biopsy”, and “combined biopsy”. Priority was given to randomized controlled trials, large prospective and retrospective cohorts, meta-analyses, and guideline documents (European Association of Urology (EAU), American Urological Association (AUA)), supplemented by manual screening of reference lists. Study selection was guided by clinical relevance to contemporary diagnostic pathways rather than by formal systematic review criteria. This framework is intended as an integrative clinical aid, not a guideline or consensus statement, and should be interpreted alongside established international recommendations. The aim of this narrative review is to synthesize contemporary evidence on PSA interpretation, mpMRI findings, biopsy strategy selection, and follow-up after negative or equivocal evaluations. We propose a practical, clinically oriented framework to optimize diagnostic precision while minimizing overdiagnosis and procedural morbidity.

## 2. Interpretation of PSA: Red Flags, False Positives, and Inflammatory Patterns

Prostate-specific antigen remains pivotal for early detection; however, its interpretation requires a nuanced, multimodal approach. Accordingly, clinically significant prostate cancer (ISUP Grade Group ≥ 2) represents the primary diagnostic target of PSA-based risk stratification. Contemporary evaluation, therefore, emphasizes PSA-derived parameters and supplementary biomarkers to enhance risk stratification for csPCa [8,9].

### 2.1. PSA Total, PSA Density, PSA Velocity: Decision Thresholds

Because total PSA alone lacks sufficient specificity, clinical decision-making increasingly relies on derived metrics, particularly PSAD and PSA velocity (PSAV), especially in patients with indeterminate imaging findings [2].

Prostate-specific antigen density, calculated as PSA divided by prostate volume, provides critical contextualization of PSA elevation. A threshold of ≥0.15 ng/mL/cc is consistently associated with csPCa and is widely used to inform biopsy decisions in men with equivocal mpMRI findings [16,17].

Although MRI-derived prostate volume is considered the gold standard for PSAD calculation due to its superior precision, clinical workflows commonly rely on transrectal ultrasound (TRUS)-measured prostate volume for initial PSAD estimation during the first urological evaluation. This preliminary PSAD is often used as a pragmatic risk-stratification tool to inform mpMRI acquisition. Following mpMRI, PSAD may be recalculated using MRI-derived prostate volume (MRI-PSAD), allowing for refined decision-making, particularly in equivocal scenarios such as PI-RADS 3 lesions [18].

Prostate-specific antigen velocity reflects the rate of change in PSA over time. A sustained increase exceeding 0.75 ng/mL/year has been associated with a higher likelihood of underlying malignancy. Although PSA velocity alone is no longer considered a standalone indication for biopsy, sustained progressive increases remain clinically informative when interpreted alongside PSA density and mpMRI findings [19]. Importantly, PSA velocity should not be used as a standalone indication for prostate biopsy but rather interpreted as a supportive parameter alongside PSA density, mpMRI findings, and overall clinical risk, in accordance with current guideline recommendations.

It should be acknowledged that both PSA density and PSA kinetics depend on parameters that may not be immediately available during a single clinical encounter. PSA density requires prostate volumetry, which in routine practice is typically obtained via TRUS at the initial urological evaluation, without mandating upfront mpMRI [16]. In contrast, PSA kinetics are inherently longitudinal and rely on serial PSA measurements over time rather than on isolated values [19,20]. While guideline recommendations often reference fixed screening intervals, such as biannual PSA testing, real-world clinical decision-making frequently integrates available PSA trends, prior values, and clinical context to inform risk stratification. Accordingly, PSA-derived metrics in this framework are intended to support flexible, context-adapted decision-making rather than function as rigid or time-dependent thresholds [2,13].

In addition, composite biomarkers such as the PHI and 4Kscore improve discrimination between benign disease and csPCa and have demonstrated value in reducing unnecessary biopsies when incorporated into diagnostic algorithms [8,9]. In clinical practice, these biomarkers are most valuable in patients with negative (PI-RADS 1–2) or equivocal (PI-RADS 3) mpMRI findings, where they may meaningfully influence decisions to defer biopsy, proceed with systematic sampling, or continue structured surveillance [2].

### 2.2. PSA Kinetics and Evolution Patterns: Progressive, Oscillatory, Wave-like, Post-Inflammatory

**Figure 1:**
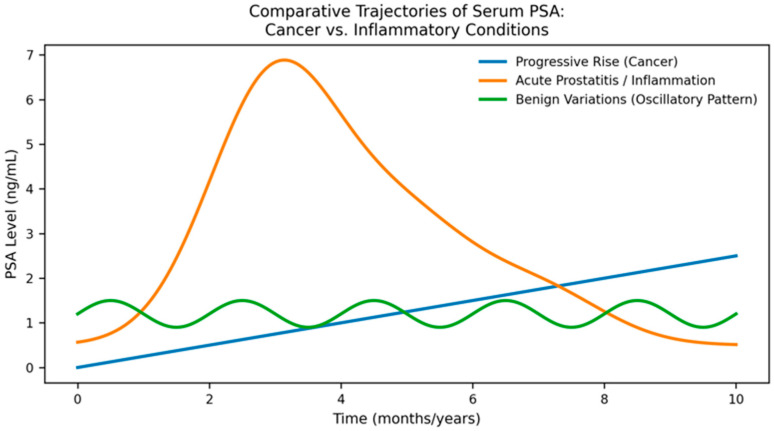
Conceptual representation of typical PSA trajectories observed in clinically significant prostate cancer, prostatitis, and benign conditions, based on commonly reported patterns in clinical practice rather than individual patient-level datasets.

In clinical practice, PSA trajectories often deviate from the classical progressive rise associated with prostate cancer. Several recurring kinetic patterns can be identified (Figure 1).

A progressive, monotonic PSA increase across serial measurements is the most concerning pattern for csPCa, particularly when accompanied by rising PSAD [20]. In contrast, oscillatory PSA fluctuations are frequently observed in inflammatory conditions or following transient prostatic insults and are less suggestive of malignancy [10]. Post-inflammatory wave-like kinetics—characterized by a sharp rise followed by gradual partial decline and stabilization over several months—are typical of prostatitis [21]. Transient PSA elevations may also occur after instrumentation, catheterization, cystoscopy, or ejaculation [22,23].

Recognition of these kinetic patterns may prevent unnecessary imaging or biopsy.

### 2.3. Distinguishing Prostatitis from Prostate Cancer Using PSA Dynamics

Prostate-specific antigen dynamics provide valuable clues for distinguishing inflammatory processes from malignancy. An abrupt increase in PSA levels, followed by a spontaneous partial decline, suggests an inflammatory process rather than malignancy [10,19]. Large PSA excursions over short intervals (≥1–2 ng/mL within weeks) are more characteristic of prostatitis than csPCa [21]. PSA disproportionate to prostate volume—high PSA in the context of a small gland—raises suspicion for malignancy, whereas high PSA associated with very large prostate volume more often reflects benign hyperplasia [8].

### 2.4. When PSA Alone Justifies Immediate mpMRI

Immediate mpMRI evaluation may be considered in several PSA-driven clinical scenarios: PSA ≥ 3 ng/mL in men at intermediate risk [13]; PSAD ≥ 0.15 ng/mL/cc regardless of absolute PSA [16,17]; a documented progressive PSA rise across two or more consecutive measurements [20]; borderline PSA values in men with a family history or genetic predisposition to prostate cancer [13]; and a digital rectal examination (DRE) suspicious for malignancy [2]. In these settings, mpMRI significantly improves the detection of csPCa while reducing unnecessary biopsies [24,25].

### 2.5. When PSA Kinetics Allow Observation Rather than Immediate Imaging

Conversely, surveillance rather than immediate mpMRI may be appropriate when PSA fluctuations are small and inconsistent without a progressive trend [20], when recent ejaculation, prostatitis symptoms, or urinary infection are present [10,22], when PSAD remains below 0.15 ng/mL/cc despite mildly elevated PSA [16], when prostate volume is large with stable PSA [6], or after both negative mpMRI and negative biopsy provided PSA does not continue to rise [24]. When PSA fluctuations are modest, inconsistent, and lack a clear progressive trend, immediate imaging rarely adds meaningful diagnostic value. In such situations, short-term observation with repeat PSA testing is often more informative than reflex mpMRI. Reassessment after 6–8 weeks, performed under standardized conditions and avoiding recent ejaculation, infection, or instrumentation, allows for transient inflammatory effects to resolve and provides a clearer signal for subsequent decision-making.

## 3. The Role of mpMRI in the Contemporary Diagnostic Pathway

The integration of mpMRI into the diagnostic pathway has substantially changed how prostate cancer risk is assessed, stratified, and managed. Instead of functioning as a standalone imaging modality, mpMRI now serves as an integral decision-making tool that refines the interpretation of PSA abnormalities, guides biopsy strategies, and influences ongoing surveillance [6,8,16].

### 3.1. Multiparametric MRI as the Diagnostic Gatekeeper: Evidence and Clinical Impact

In current clinical pathways, mpMRI often represents the point at which PSA abnormalities are translated into biopsy decisions. Its value lies less in functioning as an isolated imaging test and more in its ability to refine biopsy selection and guide how tissue is sampled. Evidence from large prospective trials consistently suggests that MRI-targeted pathways preferentially identify clinically significant disease while limiting the detection of low-risk tumors [3,4]. Subsequent systematic reviews and meta-analyses have largely confirmed these observations across different clinical settings [24,25].

Outside controlled trial settings, real-world cohorts have supported the role of mpMRI as a primary diagnostic gatekeeper. Large multicenter datasets demonstrate significant reductions in unnecessary and negative initial biopsies when MRI is incorporated early in the diagnostic process [26]. This diagnostic strategy reflects a strong convergence between international guidelines. Both the EAU and AUA recommend the use of mpMRI prior to biopsy in both biopsy-naive and prior-negative patients, with the shared aim of refining biopsy selection and improving the detection of clinically significant disease [2,13]. While there is broad alignment between international guidelines regarding the role of mpMRI, subtle differences in emphasis exist. The EAU guidelines place greater structured weight on PSA density to support biopsy avoidance in patients with negative mpMRI (PI-RADS 1–2) [16,17], whereas the AUA guidelines emphasize shared decision-making and the selective use of adjunctive biomarkers, such as the PHI or 4Kscore, to refine risk stratification when imaging findings are inconclusive [8,9,13]. Practice-based evidence has delineated the specific clinical contexts in which mpMRI performance is limited, particularly in patients with intermediate baseline risk or elevated PSA density. Depending on baseline clinical risk and PSA density, 10–30% of significant tumors may remain undetected [6,27], as emphasized in systematic evaluations of mpMRI negative predictive value [12].

### 3.2. Integrating mpMRI Findings with PSA Kinetics and Risk Stratification

The clinical relevance of mpMRI is not limited to lesion detection but also lies in how it reshapes the interpretation of PSA abnormalities. Instead of functioning as a binary trigger for biopsy, PSA becomes clinically informative only when interpreted alongside MRI findings, prostate volume, temporal PSA patterns, and complementary biomarkers [8,9].

A low PSAD in the setting of a PI-RADS 1–2 mpMRI is generally associated with benign findings and often justifies conservative management [16,17]. In contrast, progressive PSA elevation, increased PSAD, and PI-RADS 4–5 lesions substantially increase the likelihood of clinically significant disease [11,14,28]. PI-RADS 3 lesions represent a diagnostic grey zone in which imaging alone is insufficient. In this setting, biopsy decisions depend primarily on PSAD measures, PSA kinetics, inflammatory features, and individual risk factors rather than on MRI appearance in isolation [14,16]. In particular, PI-RADS 3 lesions should be managed through a dedicated, risk-adapted diagnostic pathway that incorporates PSA density, PSA kinetics, adjunctive biomarkers, and structured follow-up strategies, rather than relying on MRI appearance alone.

### 3.3. Implications for Biopsy Strategy: Targeted Sampling and the Role of Systematic Cores

The incorporation of mpMRI into the diagnostic pathway has directly altered how prostate biopsy strategies are selected, especially in men with a prior negative biopsy, where traditional transrectal sampling often fails to access anterior, apical, or transition-zone tumors adequately. Targeting lesions within these previously under-sampled regions allows mpMRI-guided biopsy to capture clinically significant tumors that are commonly missed by conventional transrectal sampling [11,28].

Although a negative mpMRI reduces the probability of clinically significant disease, it cannot be interpreted as definitive, particularly in men undergoing repeat biopsy. Follow-up studies indicate that a subset of men with negative imaging are later found to have clinically significant disease, underscoring the need for ongoing PSA-based surveillance guided by PSA density and temporal trends [6].

The method of tissue acquisition further affects diagnostic yield. MRI–ultrasound fusion biopsy is now commonly used in clinical practice, reflecting a pragmatic balance between targeting accuracy and procedural feasibility [11,28]. In-bore MRI biopsy offers maximal targeting precision but remains limited by cost and availability, while cognitive fusion is more operator-dependent [29]. Importantly, systematic sampling retains a fundamental role in the mpMRI era. A meaningful proportion of csPCa—particularly multifocal or MRI-occult tumors—is detected exclusively in systematic cores, supporting combined targeted-plus-systematic strategies endorsed by international guidelines [2,11,13].

### 3.4. Limitations and Potential Pitfalls of mpMRI in Clinical Practice

Despite its strengths, mpMRI is subject to several limitations in routine practice. Contemporary prostate mpMRI is performed using 1.5 T or 3 T scanners in accordance with PI-RADS recommendations. While higher field strength is generally associated with improved spatial resolution and lesion conspicuity, diagnostic performance remains acceptable at both levels when standardized acquisition protocols and experienced interpretation are applied [14]. Prostatitis and chronic inflammatory changes may mimic PI-RADS 3–4 lesions, generating false-positive interpretations [10,21]. Very large prostate volumes can impair lesion conspicuity. PSA fluctuations related to infection, instrumentation, or ejaculation may prompt imaging at suboptimal time points [22,23]. Inter-reader variability, particularly for intermediate-suspicion categories, remains a challenge despite refinements introduced in PI-RADS v2.1 [12,14]. In this context, emerging artificial intelligence (AI)-based tools for prostate MRI assessment warrant further consideration. Several AI-assisted platforms have been developed to support PI-RADS assessment by standardizing lesion detection, segmentation, and risk stratification. Their primary aim is to reduce inter-reader variability and improve reproducibility across different levels of radiological expertise. Early validation studies suggest that AI-assisted MRI interpretation may enhance consistency in PI-RADS scoring and improve the detection of clinically significant prostate cancer, particularly among less-experienced readers [30]. However, current evidence supports the role of AI primarily as a decision-support tool rather than a replacement for expert radiological interpretation. At present, AI-based systems should be viewed as complementary technologies that may facilitate more consistent MRI reporting and multidisciplinary communication, while prospective validation and real-world integration studies remain ongoing [31].

In parallel, there is growing interest in biparametric MRI (bpMRI) protocols that omit dynamic contrast enhancement, motivated by efforts to reduce examination time, cost, and contrast-related risks [32]. Several studies have demonstrated that bpMRI may achieve diagnostic performance comparable to multiparametric MRI for the detection of clinically significant prostate cancer in selected patient populations, particularly in biopsy-naive men and in screening or triage settings [33]. However, the incremental value of dynamic contrast enhancement remains relevant in specific clinical scenarios, including equivocal PI-RADS 3 lesions, prior negative biopsies, and post-treatment or inflammatory changes [14]. Accordingly, bpMRI should be viewed as a pragmatic alternative within resource-conscious diagnostic pathways, while mpMRI continues to play an important role in complex or indeterminate cases. These limitations underscore the need to interpret mpMRI within a broader multimodal clinical framework.

### 3.5. Conclusion: The Multimodal Assessment Framework

Multiparametric MRI now functions as a key decision-making tool within contemporary prostate cancer diagnostic pathways. It improves PSA interpretation, enhances biopsy precision, and reduces unnecessary procedures. However, its full clinical value emerges only when applied judiciously and embedded within a structured multimodal framework that integrates imaging data with PSA kinetics, prostate volume, biopsy history, and individualized patient risk [2,13].

## 4. Choosing the Biopsy Strategy: Targeted, Systematic, or Combined Sampling

**Figure 2:**
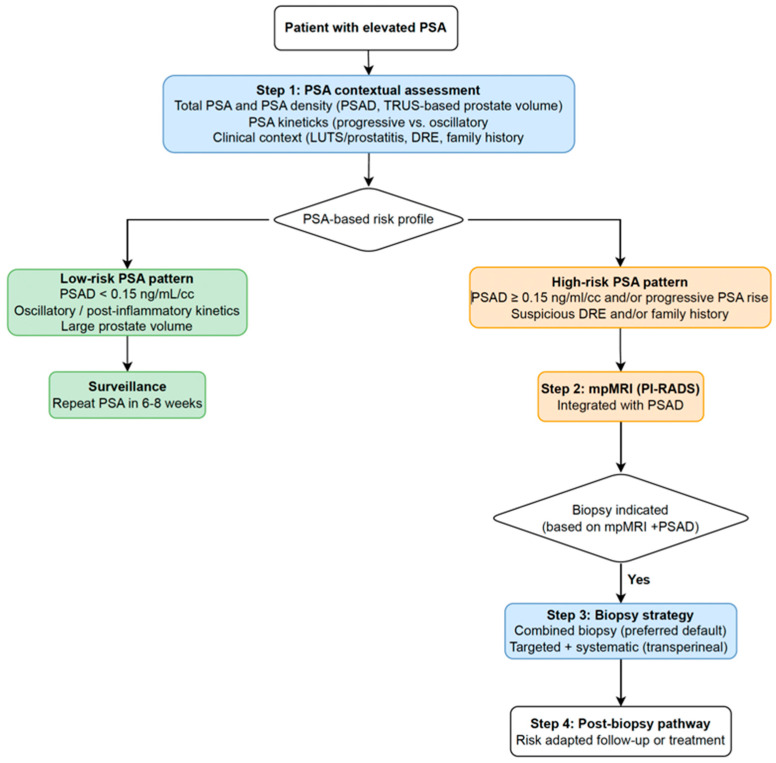
Integrated diagnostic pathway for prostate biopsy decision-making in men with elevated PSA. The algorithm combines PSA contextual assessment, PSA density, PSA kinetics, and mpMRI findings to support risk stratification and biopsy strategy selection. This pathway represents a conceptual, expert-derived framework intended to aid clinical decision-making and has not been prospectively validated. Its application should be interpreted in conjunction with established international guidelines and the individual patient context.

The transition toward mpMRI-driven diagnostic pathways has fundamentally shifted attention to how prostate tissue should be sampled once imaging results are available [3,4]. The optimal biopsy strategy is not uniform but depends on lesion visibility, PSAD, prior biopsy history, and overall clinical suspicion [6,16,17]. In contemporary practice, clinicians navigate three principal approaches—targeted, systematic, and combined biopsy—each with distinct indications and diagnostic trade-offs [34]. Representative evidence from key prospective trials comparing systematic, MRI-targeted, and combined biopsy strategies is summarized in Table 1, while an integrated diagnostic pathway that combines PSA contextual assessment, PSA density, mpMRI findings, and biopsy strategy selection is illustrated in Figure 2. Key clinical take-home messages derived from the above considerations are summarized in Box 1.

Box 1 **Key Clinical Take-Home Messages.**

• PSA should not be interpreted in isolation during clinical decision-making.
  PSA density, PSA kinetics, prostate volume, and clinical context are essential for accurate risk stratification and for avoiding unnecessary imaging or biopsy.
• mpMRI functions as a diagnostic gatekeeper, not a standalone test.
  While mpMRI substantially improves the detection of clinically significant prostate cancer, negative imaging does not definitively exclude disease, particularly in patients with elevated PSA density or progressive PSA kinetics.
• PSA density ≥ 0.15 ng/mL/cc represents a pivotal decision anchor across diagnostic scenarios.
  This threshold consistently refines biopsy indications in patients with negative or equivocal mpMRI findings.
• Combined targeted and systematic biopsy currently offers the most robust diagnostic performance.
  For most biopsy-naive and high-risk patients, combined sampling maximizes detection of clinically significant cancer while limiting clinically irrelevant overdiagnosis.
• Grey-zone scenarios benefit from integrated, longitudinal assessment rather than immediate or uncontextualized intervention.
  Oscillatory PSA patterns, inflammatory features, and repeated negative evaluations should prompt structured surveillance rather than repeated immediate biopsy.

### 4.1. Targeted (MRI–Ultrasound Fusion) Biopsy: When It May Serve as a First-Line Option

MRI-targeted biopsy, performed via software-based fusion, cognitive targeting, or in-bore guidance, is generally considered an appropriate strategy when mpMRI identifies a clearly suspicious lesion. By leveraging the spatial resolution of mpMRI, targeted sampling consistently improves the detection of csPCa compared with systematic biopsy alone [11,28].

Targeted biopsy is the primary approach in many scenarios. PI-RADS 4–5 lesions have a high pre-test probability of csPCa and derive the most significant incremental benefit from targeted cores [11,14,28]. In PI-RADS 3 lesions, PSAD thresholds—particularly when PSA density exceeds accepted risk thresholds—play a decisive role in selecting patients for biopsy [16,17]. Targeted biopsy is also particularly valuable in men undergoing repeat biopsy after a prior negative result, as mpMRI frequently reveals anterior, apical, or transition-zone tumors missed by conventional transrectal sampling [11,28]. Similarly, lesions located in anatomically challenging regions benefit from fusion guidance, where systematic sampling has reduced accuracy [35].

Although targeted-only biopsy offers excellent diagnostic yield when lesions are well defined, its performance is intrinsically dependent on lesion visibility and targeting accuracy. For this reason, its use as a standalone strategy remains selective rather than routine.

### 4.2. Systematic Biopsy: When It Remains Necessary in the mpMRI Era

Despite the transformative effect of mpMRI, systematic biopsy remains a crucial complementary role. Contemporary evidence indicates that exclusive reliance on MRI-targeted sampling may overlook clinically significant disease, particularly in cases of multifocal growth patterns, small-volume tumors, or lesions located outside the dominant MRI-visible focus [36]. In this context, systematic cores ensure comprehensive glandular sampling that is not contingent on MRI conspicuity alone.

In patients with negative mpMRI findings, guideline recommendations emphasize risk-adapted decision-making rather than uniform biopsy avoidance. The EAU guidelines place particular emphasis on PSA density, supporting biopsy omission in selected patients with low PSAD and PI-RADS 1–2 imaging [2]. In contrast, the AUA guidelines adopt a similar risk-based approach but place greater emphasis on shared decision-making, incorporating patient preferences and, when appropriate, the selective use of adjunctive biomarkers to further refine residual risk [13].

Systematic biopsy remains indicated in several clinical situations. Men with negative mpMRI but elevated PSAD or persistent clinical suspicion warrant systematic sampling, as low-suspicion imaging reduces, but does not eliminate, the likelihood of csPCa [6,16,17]. A suspicious DRE supersedes imaging findings and mandates biopsy regardless of mpMRI results [2,13]. Systematic cores are also critical when multifocal disease is suspected, given MRI’s known limitations in fully characterizing tumor burden [11,12]. Finally, high-risk screening populations, including those with hereditary predisposition or germline mutations, may harbor early or MRI-inconspicuous disease, supporting the continued role of systematic sampling [2,13].

### 4.3. Combined Biopsy (Targeted + Systematic): The Contemporary Diagnostic Standard

Extensive comparative studies consistently demonstrate that combining targeted and systematic biopsy yields the highest diagnostic accuracy for csPCa [11,28]. This dual approach capitalizes on the strengths of each technique: targeted cores efficiently sample MRI-visible high-grade lesions. In contrast, systematic cores detect additional clinically significant disease outside the index lesion [11,37]. In many contemporary centers, combined biopsy is increasingly performed via the transperineal approach, which offers more comprehensive glandular sampling and a substantially lower risk of infectious complications compared with the transrectal route and has consequently become the preferred access for systematic and combined prostate biopsy [2].

Combined biopsy is now widely regarded as an appropriate strategy for most biopsy-naive patients. It achieves maximal sensitivity, mitigates the limitations of mpMRI—including inter-reader variability and lesion underestimation—and improves overall risk stratification without a disproportionate increase in overdiagnosis [12,14]. While systematic sampling may increase the detection of Gleason 6 tumors, contemporary data suggest that this incremental rise is modest and does not translate into a clinically meaningful increase in overtreatment when systematic cores are performed alongside high-yield targeted sampling [37].

Accordingly, both European and American guidelines endorse combined biopsy as the default diagnostic strategy [2,13]. Targeted-only pathways are reserved for narrow, highly selected scenarios, such as solitary, highly conspicuous PI-RADS 5 lesions with elevated PSAD [38], patients undergoing in-bore biopsy [29], or repeat biopsy after extensive prior systematic sampling [36]. Outside these exceptions, combined sampling represents the most widely adopted diagnostic approach in contemporary practice.

## 5. Special Clinical Scenarios: Prostatitis, Oscillatory PSA Profiles, and Repeated Negative Evaluations

A substantial proportion of diagnostic uncertainty in contemporary prostate cancer assessment arises not from clearly malignant findings but from “grey zone” clinical scenarios. These include patients with fluctuating PSA values, chronic pelvic or urinary symptoms, or persistently abnormal PSA despite negative imaging and biopsy results. In such situations, PSA kinetics, mpMRI findings, and clinical context must be interpreted as an integrated whole rather than in isolation. Recognizing characteristic inflammatory patterns and non-malignant PSA behavior is essential to avoid unnecessary biopsies while ensuring that csPCa is not overlooked.

### 5.1. When Elevated PSA Signals Prostatitis Rather than Cancer

Prostatic inflammation, whether acute or chronic, frequently causes PSA elevations that mimic malignancy. Several features favor prostatitis over csPCa. A typical pattern is PSA elevation disproportionate to prostate volume, resulting in a relatively low PSAD despite markedly increased PSA values [8,10,21]. PSA levels may also exhibit large oscillations over short intervals, a behavior uncommon in untreated csPCa but typical of inflammatory activity [10,21]. Clinically, pelvic or perineal discomfort, fluctuating lower urinary tract symptoms, or mild elevations in inflammatory markers further support a non-malignant etiology [21].

On mpMRI, prostatitis often manifests as diffuse or ill-defined inflammatory changes, including T2 hypointensity, diffuse apparent diffusion coefficient reduction, or patchy enhancement, without a focal lesion conforming to PI-RADS criteria [14,21]. Without careful clinical correlation, these findings may be misinterpreted as PI-RADS 3–4 lesions.

Although normalization of PSA following antibiotic or nonsteroidal anti-inflammatory therapy may support an inflammatory etiology in symptomatic patients, routine empirical antibiotic treatment in asymptomatic men with isolated PSA elevation is generally discouraged [21]. A more appropriate approach is to repeat PSA testing after 6–8 weeks, avoiding ejaculation, cycling, urinary infection, or instrumentation beforehand [10,22]. Repeat mpMRI should be reserved for persistent PSA abnormalities, rising PSAD, or newly concerning DRE findings [14].

### 5.2. Oscillatory and Wave-like PSA Patterns

Not all PSA elevations follow a progressive, malignancy-associated trajectory. Many patients exhibit oscillatory or wave-like PSA kinetics, characterized by alternating rises and declines over time. These patterns are far more typical of inflammatory processes—either overt or subclinical—than of csPCa [10,19]. Following inflammatory episodes, PSA may take several months to stabilize, resulting in prolonged wave-like behavior [21].

When PSA variability lacks a clear progressive trend, and PSAD remains within low-risk ranges, surveillance with structured PSA monitoring is generally preferred over immediate biopsy or mpMRI [16,17,21].

### 5.3. Persistently Elevated PSA Despite Negative Biopsy

Persistent PSA elevation following a negative biopsy represents a frequent clinical challenge. In this context, histopathologic findings from the initial biopsy are critical. A diagnosis of atypical small acinar proliferation (ASAP) is associated with a substantial likelihood of detecting csPCa on repeat sampling, justifying re-biopsy within 6–12 months [15]. Multifocal high-grade prostatic intraepithelial neoplasia also warrants close surveillance and consideration of repeat biopsy, although its predictive value is lower than that of ASAP [15].

Adjunctive biomarkers may further refine risk stratification in men with persistent clinical suspicion despite negative histology, improving the discrimination of clinically significant disease and helping to reduce unnecessary repeat biopsies [8,9,34]. Emerging urinary assays have shown particular value in identifying high-grade cancer even when imaging findings are negative or equivocal [39].

From an imaging perspective, a negative mpMRI substantially lowers—but does not eliminate—the likelihood of clinically significant prostate cancer. Repeat imaging is primarily justified when PSA kinetics evolve from oscillatory to progressive patterns, PSA density increases, or new abnormalities are detected on DRE [12,14].

### 5.4. Repeatedly Negative mpMRI and Biopsy: When Suspicion Persists

A small subset of patients exhibit persistent PSA abnormalities despite repeated negative mpMRI and biopsy results. In these cases, clinical decision-making must integrate PSA trends, PSAD, genetic or familial risk factors—including BRCA1/2, ATM, or HOXB13 mutations—and biopsy technique [2,13]. A transperineal approach with extended or saturation templates offers more comprehensive gland coverage and improves the detection of anterior or apical tumors that may be missed by transrectal biopsy [11,35].

In men with ongoing clinical suspicion, a combined targeted-plus-systematic transperineal biopsy remains the most comprehensive diagnostic strategy.

## 6. Limitations and Future Directions

This narrative review presents several inherent limitations that warrant acknowledgment. First, as a non-systematic literature review, it does not adhere to a formal meta-analytical methodology and may consequently be susceptible to selection bias. Although the evidence discussed is derived from high-quality randomized trials, meta-analyses, and contemporary real-world cohorts, substantial heterogeneity exists across study populations, imaging protocols, biopsy techniques, and outcome definitions. This variability inevitably constrains direct comparability among studies [24,25]. Specifically, variability in mpMRI acquisition quality, reader expertise, and PI-RADS interpretation continues to influence diagnostic performance and the generalizability of findings [12,14].

Secondly, parameters based on PSA and emerging biomarkers, despite their growing incorporation into clinical practice, remain affected by biological variability and non-malignant conditions. Thresholds such as PSA density cut-offs or PSA kinetic patterns, while clinically valuable, should not be regarded as definitive decision criteria [16,17]. Additionally, the availability of advanced biomarkers and mpMRI remains inconsistent across various healthcare systems, which may constrain the implementation of integrated diagnostic pathways in resource-limited settings [2,13].

Thirdly, the biopsy strategies discussed in this review reflect evolving standards of care. While combined targeted and systematic biopsy currently constitutes the most robust diagnostic approach for the majority of patients, the optimal balance between diagnostic yield and overdiagnosis remains subject to ongoing refinement [11,28]. Long-term oncologic outcomes associated with targeted-only or reduced-core strategies remain inadequately characterized [37,38].

Future research should focus on prospective validation of integrated risk-adapted diagnostic algorithms that combine PSA dynamics, mpMRI findings, biomarkers, and clinical risk factors [6,34]. Within this framework, advances in artificial intelligence-assisted MRI interpretation, standardized reporting, and quantitative imaging metrics hold promise for reducing inter-reader variability and improving lesion characterization [31]. Additionally, further studies evaluating the safety of biopsy de-escalation strategies in carefully selected patient populations are needed [36,38]. Ultimately, individualized, data-driven diagnostic pathways represent the most promising direction for optimizing prostate cancer detection while minimizing unnecessary interventions.

## 7. Conclusions

Contemporary prostate cancer diagnostics require a shift from isolated decision-making toward an integrated, risk-adapted approach. Diagnostic accuracy improves when PSA dynamics and PSA density are interpreted alongside mpMRI findings and an appropriately selected biopsy strategy, rather than being considered in isolation. This multimodal framework allows for improved detection of clinically significant disease while minimizing unnecessary biopsies and diagnostic uncertainty.

Importantly, no diagnostic modality is infallible. Limitations related to equivocal imaging findings, inflammatory conditions, and MRI-occult or multifocal disease underscore the need to carefully integrate imaging results with PSA kinetics, prostate volume, and individual risk profiles when selecting patients for imaging, biopsy, and follow-up.

With respect to tissue sampling, combined targeted and systematic biopsy is currently associated with the most robust diagnostic performance for most biopsy-naive and high-risk patients, balancing sensitivity and diagnostic safety. While targeted-only or de-escalated biopsy strategies may be appropriate in carefully selected patients, these approaches should remain exceptions rather than routine practice.

Ultimately, structured, multimodal diagnostic pathways that integrate biomarkers, advanced imaging, and clinical judgment offer the most effective means of optimizing prostate cancer detection while minimizing overdiagnosis and procedural morbidity. Ongoing prospective validation and technological advances, including artificial intelligence-assisted imaging, are expected to refine risk stratification further and personalize diagnostic decision-making in the PSA era.

While the multimodal, risk-adapted diagnostic approach outlined in this review is supported by the current body of evidence, it is important to acknowledge the emergence of alternative paradigms. In particular, an initial triage strategy based on PSA assessment combined with bpMRI has been proposed and increasingly evaluated as a pragmatic approach in selected clinical settings, offering potential reductions in imaging time, costs, and resource utilization. Such strategies may be appropriate for population-level screening or initial risk stratification, whereas comprehensive multiparametric MRI and integrated multimodal assessment remain particularly important in complex, equivocal, or high-risk clinical scenarios.

## Figures and Tables

**Table 1:** Comparative diagnostic yield of systematic, MRI-targeted, and combined prostate biopsy strategies in key prospective trial.

Study	Population	Biopsy Strategies Compared	csPCa Detection—Key Findings	Main Conclusion
PROMIS (2017) [3]	Biopsy–naive	TRUS systematic vs. mpMRI + mapping biopsy	mpMRI sensitivity for csPCa 93%; systematic TRUS missed significant cancers	mpMRI improves detection but does not eliminate need for sampling
PRECISION (2018) [4]	Biopsy–naive	MRI-targeted vs. systematic TRUS	Higher csPCa detection with targeted biopsy; fewer low-risk cancers	MRI-targeted biopsy was associated with improved detection compared with systematic biopsy alone
MRI-FIRST (2019) [28]	Biopsy–naive	Targeted vs. systematic vs. combined	Combined biopsy detected more csPCa than either strategy alone	Combined biopsy provided the highest overall diagnostic yield
Ahdoot et al. (2020) [11]	Biopsy–naive	Targeted vs. systematic vs. combined	Combined biopsy detected additional csPCa missed by targeted-only	Systematic sampling remains complementary to MRI-targeted biopsy

csPCa: clinically significant prostate cancer (ISUP Grade Group ≥ 2).

## Data Availability

No new data were created or analyzed in this study. Data sharing is not applicable to this article.

## Abbreviations

The following abbreviations are used in this manuscript:

mpMRI	Multiparametric Magnetic Resonance Imaging
PSA	Prostate-Specific Antigen
csPCa	Clinically Significant Prostate Cancer
MRI	Magnetic Resonance Imaging
ISUP	International Society of Urological Pathology
PSAD	Prostate-Specific Antigen Density
PHI	Prostate Health Index
PI-RADS	Prostate Imaging Reporting and Data System
TRUS	Transrectal Ultrasound
PSAV	Prostate-Specific Antigen Velocity
DRE	Digital Rectal Examination
EAU	European Association of Urology
AUA	American Urological Association
ASAP	Atypical Small Acinar Proliferation
HGPIN	High-Grade Prostatic Intraepithelial Neoplasia
AI	Artificial Intelligence
